# Association between Serum Uric Acid and Nonalcoholic Fatty Liver Disease in Nonobese Postmenopausal Women: A Cross-sectional Study

**DOI:** 10.1038/s41598-020-66931-9

**Published:** 2020-06-22

**Authors:** Ting Bao, Zhiye Ying, Li Gong, Jing Du, Guiyi Ji, Zhengzheng Li, Wei Gao, Xianweng Jiang, Hanwei Yang, Yan Huang, Huairong Tang

**Affiliations:** 1 Health Management Center, West China Hospital, Sichuan University, Chengdu, China; 2 West China Biomedical Big Data Centre, West China Hospital, Sichuan University, Chengdu, China; 3 Outpatient department, West China Hospital, Sichuan University, Chengdu, China

**Keywords:** Medical research, Risk factors

## Abstract

This study aimed to determine the association between serum uric acid (sUA) and nonalcoholic fatty liver disease (NAFLD) in nonobese postmenopausal women. A total of 4323 female individuals over 18 years of age participated in this cross-sectional study. The subjects were divided into four groups according to menopause status and body mass index. sUA quartiles in this female population were categorized as follows: Q1 ≤ 230 mmol/L, Q2: 231–270 mmol/L, Q3: 271–310 mmol/L and Q4: ≥ 311 mmol/L. The presence or absence of NAFLD was assessed by abdominal ultrasonography. The prevalence of NAFLD was 38.8% in the general population, and the average age was 46.5 ± 11.3 years. Among nonobese and obese subjects, the prevalence of NAFLD was lower in nonmenopausal subjects than in postmenopausal subjects (nonobese: 20.74% vs 45.26%, respectively, P < 0.0001; obese: 70.51% vs 84.35%, respectively, P < 0.0001). After adjusting for age, current smoking status, current alcohol drinking status, diabetes, hypertension disease and triglyceride, the ORs (95% CIs) for NAFLD among individuals in Q2-Q4 were 1.518 (1.062–2.169), 1.431 (1.010–2.027) and 2.054 (1.442–2.927), respectively, *P* value for trend <0.0001. Higher sUA levels can be used as a predictive biomarker for NAFLD in nonobese postmenopausal women.

## Introduction

Nonalcoholic fatty liver disease (NAFLD) is recognized as a major cause of liver disease worldwide^1^. A number of studies have suggested that metabolic comorbidities, such as obesity, type 2 diabetes, hyperlipidemia, hypertension and metabolic syndrome, are important risk factors for the development of NAFLD^2–5^. The prevalence of NAFLD in the general population of China is 25–31%^6,7^.

It is well established that NAFLD is closely associated with serum uric acid (sUA)^8^, especially in obese people^9^. The available data show that the prevalence of NAFLD may reach 41.4–61.2% among Chinese people with obesity^7^. Furthermore, males present a higher prevalence (15.68–22.88%) than females (11.42–16.61%) over 55 years of age^7,10^. A large number of studies have indicated that the association between sUA and NAFLD is significantly greater in females than in males^11,12^.

Although growing evidence suggests that the NAFLD-sUA relation is present in obese populations^13–15^, few studies have demonstrated this relationship in nonobese postmenopausal women. Especially in China, where the population is generally less obese, the prevalence of NAFLD are increasing^7^.

Therefore, it is necessary to identify the natural course of NAFLD and the factors contributing to the development of NAFLD in nonobese postmenopausal female populations, as well as to evaluate whether there are differences in the risk factors for NAFLD among sUA levels in nonobese and obese postmenopausal female populations.

In this study, we first determined the prevalence of NAFLD in groups according to the sUA quartiles and the prevalence of NAFLD in nonobese and obese female populations according to the menopause status. Second, we investigated whether the risk factors for NAFLD varied among the sUA quartiles. (Table 1)

**Table 1:** Study participants’ characteristics according to serum uric acid levels quartiles.

	Quartiles of serum uric acid levels(umol/L)				P value
	Q1	Q2	Q3	Q4	
Number	1130	1065	1070	1058	
Age	45.24 ± 10.62	45.22 ± 10.71	46.92 ± 11.11	48.74 ± 12.24	<0.0001
Wasit(cm)	73.18 ± 7.68	74.62 ± 7.93	76.36 ± 8.47	79.51 ± 9.18	<0.0001
BMI(kg/m^2^)	21.75 ± 2.54	22.14 ± 2.73	22.79 ± 2.95	23.87 ± 3.28	<0.0001
Smoking(current/ex-smoker/never)%	2.57/0.09/97.35	2.16/0.19/97.65	2.06/0.19/97.76	2.55/0.28/97.16	0.9094
Alcohol drinking(current/ex-drinker/never)%	11.59/0.18/88.23	12.77/0.09/87.14	9.91/0.00/90.09	12.29/0.09/87.62	0.2520
Systolic blood pressure(mmHg)	111.95 ± 15.88	113.32 ± 16.27	115.15 ± 17.06	119.38 ± 18.11	<0.0001
Diastolic blood pressure(mmHg)	68.41 ± 9.10	69.58 ± 9.75	70.18 ± 10.10	72.06 ± 10.65	<0.0001
Fasting plasma glucose(mmol/L)	4.99 ± 1.01	4.97 ± 0.83	5.05 ± 0.90	5.25 ± 1.10	<0.0001
Total bilirubin(umol/L)	12.70 ± 4.74	12.91 ± 5.07	13.13 ± 5.28	12.73 ± 4.91	0.1667
Direct bilirubin(umol/L)	3.58 ± 1.44	3.61 ± 1.45	3.64 ± 1.59	3.51 ± 1.45	0.2222
Indirect bilirubin(umol/L)	9.13 ± 3.57	9.30 ± 3.88	9.49 ± 3.98	9.22 ± 3.72	0.1594
HDL(mmol/L)	1.68 ± 0.38	1.62 ± 0.37	1.57 ± 0.35	1.47 ± 0.37	<0.0001
LDL(mmol/L)	2.69 ± 0.72	2.78 ± 0.79	2.90 ± 0.79	3.00 ± 0.82	<0.0001
Triglyceride(mmol/L)	1.05 ± 0.67	1.17 ± 0.88	1.27 ± 0.74	1.58 ± 1.02	<0.0001
Total cholesterol(mmol/L)	4.71 ± 0.86	4.79 ± 0.92	4.90 ± 0.92	5.01 ± 0.94	<0.0001
AST(uL)	22.32 ± 12.21	22.39 ± 8.79	22.87 ± 8.97	24.51 ± 12.00	0.0044
ALT(uL)	17.79 ± 29.71	19.11 ± 13.72	20.05 ± 14.03	22.83 ± 17.04	<0.0001
ALP(uL)	67.65 ± 24.02	71.12 ± 33.26	74.06 ± 30.78	78.43 ± 24.13	<0.0001
GGT(uL)	16.03 ± 18.74	20.38 ± 35.96	21.50 ± 28.22	26.89 ± 32.68	<0.0001
Diabetes mellitus%	1.86	2.16	1.87	4.82	<0.0001
Hypertension%	3.81	5.35	7.10	12.19	<0.0001
Non-alcoholic fatty liver disease(yes),%	25.31	32.58	41.03	57.18	<0.0001

## Results

### Study population characteristics

In the cross-sectional population, 4323 female individuals were enrolled. The prevalence of NAFLD was 38.8% in the general population, and the average age was 46.5 ± 11.3 years. Baseline characteristics of the subjects based on sUA quartile were presented in Table 1. Subjects with higher sUA levels were more likely to have a higher prevalence of NAFLD (Q1: 25.31%, Q2: 32.58%, Q3: 41.03%, Q4: 57.18%) and a higher prevalence of diabetes mellitus and hypertension. These subjects were older and had significantly greater mean waist circumference, body mass index (BMI), systolic blood pressure (SBP), diastolic blood pressure (DBP), fasting plasma glucose (FPG), low density lipoprotein cholesterol (LDL-C), triglyceride (TG), total cholesterol (TC), aspartate aminotransferase (AST), alanine aminotransferase (ALT), alkaline phosphatase (ALP), and gamma-glutamyl transpeptidase (GGT) values (Table 2).

**Table 2:** The prevalence of NAFLD and study population’ characteristics according to BMI and menopausal subgroup.

	Nonobese (n = 3490)			Obese (n = 833)		
	Non-menopausal	Postmenopausal	P value	Non-menopausal	Postmenopausal	P value
Number	2257	1233		391	483	
Age	39.22 ± 7.45	56.45 ± 7.28	<0.0001	43.10 ± 6.69	58.38 ± 8.16	<0.0001
Wasit(cm)	71.43 ± 6.05	76.22 ± 6.47	<0.0001	84.84 ± 6.16	88.58 ± 7.05	<0.0001
BMI(kg/m^2^)	21.18 ± 1.97	22.07 ± 1.81	<0.0001	26.97 ± 1.92	27.30 ± 1.97	0.0147
Smoking(current/ex-smoker/never)%	3.46/0.22/96.32	0.89/0.08/99.03	<0.0001	2.68/0.27/97.05	0.43/0.22/99.35	0.0117
Alcohol drinking(current/ex-drinker/never)%	13.51/0.04/86.44	7.79/0.16/92.05	<0.0001	16.89/0.00/83.11	8.48/0.22/91.30	0.0003
Systolic blood pressure(mmHg)	107.96 ± 12.83	121.23 ± 17.60	<0.0001	118.05 ± 16.36	128.86 ± 18.12	<0.0001
Diastolic blood pressure(mmHg)	67.43 ± 8.93	71.78 ± 10.15	<0.0001	73.08 ± 10.01	75.38 ± 10.54	0.0015
Fasting blood glucose(mmol/L)	4.82 ± 0.61	5.28 ± 1.08	<0.0001	5.22 ± 1.27	5.56 ± 1.40	0.0002
Total bilirubin(umol/L)	12.95 ± 5.29	13.07 ± 4.70	0.5010	11.56 ± 4.70	12.96 ± 4.43	<0.0001
Direct bilirubin(umol/L)	3.71 ± 1.57	3.49 ± 1.39	<0.0001	3.25 ± 1.35	3.49 ± 1.29	0.0088
Indirect bilirubin(umol/L)	9.24 ± 3.96	9.59 ± 3.61	0.0097	8.32 ± 3.57	9.48 ± 3.44	<0.0001
HDL(mmol/L)	1.62 ± 0.36	1.63 ± 0.40	0.2936	1.38 ± 0.31	1.43 ± 0.32	0.0298
LDL(mmol/L)	2.62 ± 0.72	3.13 ± 0.80	<0.0001	2.87 ± 0.70	3.14 ± 0.82	<0.0001
Triglyceride(mmol/L)	1.05 ± 0.69	1.40 ± 0.86	<0.0001	1.53 ± 1.03	1.73 ± 1.11	0.0059
Total cholesterol(mmol/L)	4.58 ± 0.83	5.25 ± 0.92	<0.0001	4.77 ± 0.82	5.19 ± 0.92	<0.0001
AST(uL)	21.20 ± 11.15	25.31 ± 23.93	<0.0001	22.75 ± 10.43	25.99 ± 10.07	<0.0001
ALT(uL)	17.50 ± 17.01	21.09 ± 25.09	<0.0001	23.41 ± 18.94	25.81 ± 16.29	0.0500
ALP(uL)	62.89 ± 18.25	87.32 ± 37.33	<0.0001	70.79 ± 21.47	88.74 ± 23.14	<0.0001
GGT(uL)	17.07 ± 21.46	23.44 ± 37.41	<0.0001	27.24 ± 35.90	30.02 ± 32.79	0.2451
Diabetes mellitus%	0.35	5.35	<0.0001	2.41	6.96	0.0026
Hypertension%	1.42	12.73	<0.0001	4.56	21.52	<0.0001
Non-alcoholic fatty liver disease(no/yes),%	20.74	45.26	<0.0001	70.51	84.35	<0.0001

### Prevalence of NAFLD and study population characteristics according to BMI and menopause status

**Figure 1:**
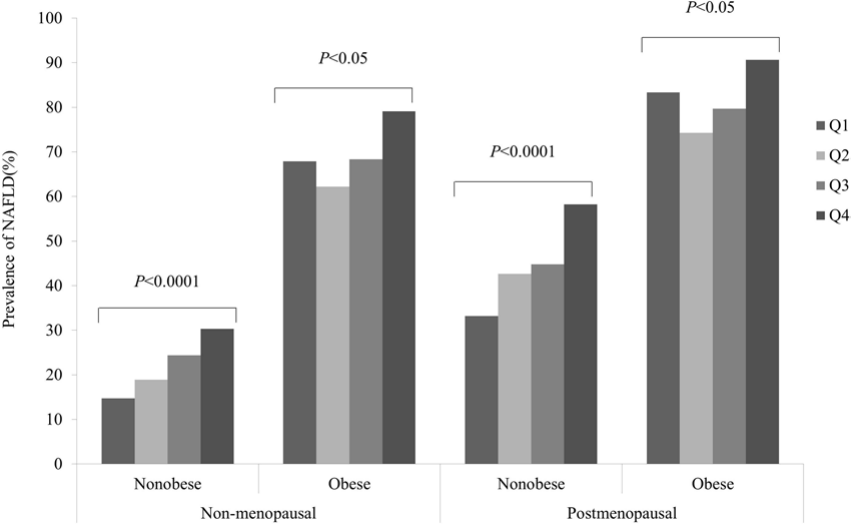
The prevalence of NAFLD according to serum uric acid levels quartiles in postmenopausal women. Q1 ≤ 230 mmol/L, Q2: 231–270 mmol/L, Q3: 271–310 mmol/L and Q4 ≥ 311 mmol/L.

**Figure 2:**
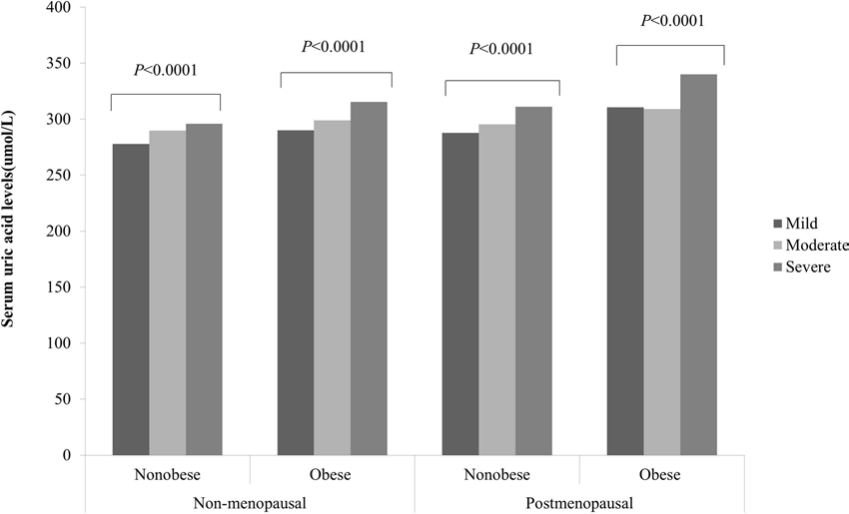
Serum uric acid levels of postmenopausal women patients according to grades of NAFLD.

**Figure 3:**
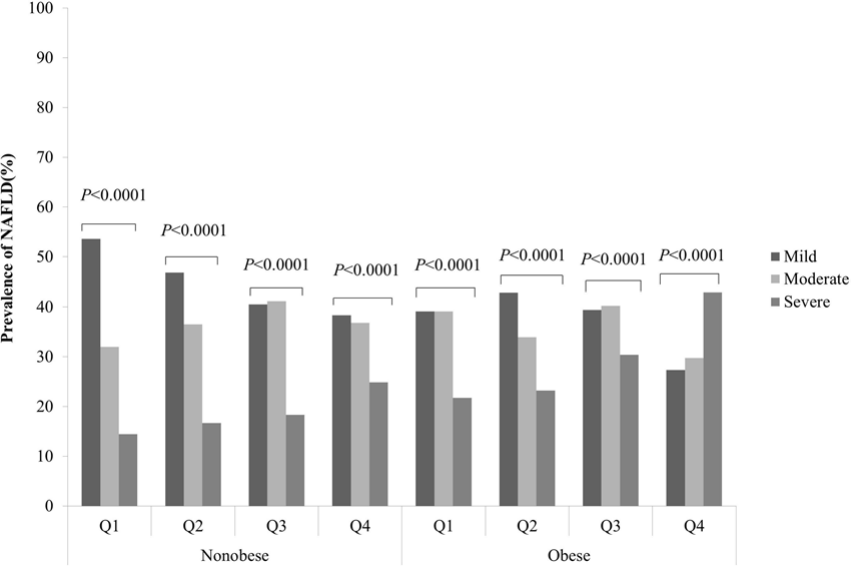
The prevalence of different grades of NAFLD according to serum uric acid levels quartiles in postmenopausal women. Q1 ≤ 230 mmol/L, Q2: 231–270 mmol/L, Q3: 271–310 mmol/L and Q4 ≥ 311 mmol/L (Table 3).

Further, we determined the prevalence of NAFLD in nonmenopausal and postmenopausal subjects in the nonobese and obese populations. Regardless of whether the subjects were nonobese or obese, the prevalence of NAFLD was lower in nonmenopausal subjects than in postmenopausal subjects (nonobese: 20.74% vs 45.26%, respectively, P < 0.0001; obese: 70.51% vs 84.35%, respectively, P < 0.0001). Among these postmenopausal subjects, older subjects had significantly higher mean waist circumference, SBP, DBP, FPG, IBIL, LDL, TG, TC, AST, ALT, and ALP values than nonmenopausal subjects in both the nonobese and obese populations. Moreover, among both nonobese and obese subjects, the prevalence of diabetes mellitus and hypertension was higher in postmenopausal subjects than in nonmenopausal subjects (Figures 1–3).

**Table 3:** The baseline characteristics according to the presence of NAFLD in postmenopausal women.

	Postmenopausal women (n = 1693)		
	Without of NAFLD	With of NAFLD	P value
Number	747	946	
Age	55.85 ± 7.26	57.87 ± 7.70	<0.0001
Wasit(cm)	75.13 ± 7.13	83.31 ± 8.02	<0.0001
BMI(kg/m2)	22.03 ± 2.35	24.74 ± 2.90	<0.0001
Smoking(current/ex-smoker/never)%	0.96/0.00/99.06	0.63/0.21/99.15	0.5056
Alcohol drinking(current/ex-drinker/never)%	8.17/0.27/91.57	7.82/0.11/92.07	0.7113
Systolic blood pressure(mmHg)	119.39 ± 17.83	126.45 ± 17.64	<0.0001
Diastolic blood pressure(mmHg)	71.03 ± 10.35	74.14 ± 10.20	<0.0001
Fasting blood glucose(mmol/L)	5.13 ± 0.92	5.53 ± 1.33	<0.0001
Total bilirubin(umol/L)	13.14 ± 4.76	12.97 ± 4.51	0.4353
Direct bilirubin(umol/L)	3.52 ± 1.39	3.47 ± 1.34	0.5363
Indirect bilirubin(umol/L)	9.63 ± 3.67	9.51 ± 3.47	0.4846
HDL(mmol/L)	1.71 ± 0.40	1.48 ± 0.35	<0.0001
LDL(mmol/L)	3.06 ± 0.79	3.18 ± 0.80	0.0021
Triglyceride(mmol/L)	1.20 ± 0.56	1.72 ± 1.11	<0.0001
Total cholesterol(mmol/L)	5.20 ± 0.92	5.27 ± 0.91	0.1332
AST(uL)	25.27 ± 29.06	25.68 ± 11.33	0.6893
ALT(uL)	19.87 ± 28.84	24.37 ± 17.06	<0.0001
ALP(uL)	85.25 ± 38.17	89.65 ± 30.27	0.0085
GGT(uL)	21.25 ± 37.96	28.38 ± 34.67	<0.0001
Diabetes mellitus%	4.28	6.98	0.0185
Hypertension%	10.98	18.39	<0.0001.

### Stratified analysis according to menopause status and BMI

Whether subjects were nonmenopausal or menopausal, in the BMI-stratified analysis, the positive association and dose-response relationship between the sUA level and the prevalence of NAFLD were significant in the nonobese population (P < 0.0001), and except for the Q1 group, with increasing sUA, the prevalence of NAFLD increased in the obese population (Fig. 1).

In addition, subjects with NAFLD were divided into three groups according to the results of abdominal ultrasonography. Whether subjects were nonmenopausal or menopausal, in the BMI-stratified analysis, the positive association and dose-response relationship between the sUA level and the severity of liver disease in individuals with NAFLD were significant in the nonobese population (P < 0.0001) (Fig. 2).

Figure 3 displays a BMI-stratified analysis of changes in the prevalence of NAFLD of varying severity according to the sUA quartiles in postmenopausal women. The correlation between elevated sUA and reduced NAFLD severity risk was more remarkable in nonobese individuals than in obese individuals. However, in the obese individuals, the risk of severe NAFLD showed an inverse trend compared with the Q1-Q3 groups (Fig. 3).

### The baseline characteristics according to the presence of NAFLD in postmenopausal women

There were 1693 postmenopausal female individuals. And the prevalence of NAFLD was 55.9%(946). Baseline characteristics of the subjects based on wether diagnosed with NAFLD were presented in Table 3. These subjects dignosed with NAFLD were older and had significantly higher mean waist circumference, BMI, SBP, DBP, FPG, LDL-C, TG, ALT, ALP, and GGT values, and more likely to have a higher prevalence of diabetes mellitus and hypertension (Table 4).

**Table 4:** Individual association of serum uric acid and non-alcoholic fatty liver disease in postmenopausal women.

SUA(umol/L)	Nonobese			Obese		
	Model1	Mode2	Model3	Model1	Model2	Model3
Q1	reference	reference	reference	reference	reference	reference
Q2	1.527(1.084–2.152)	1.514(1.074–2.133)	1.518(1.062–2.169)	0.616(0.249–1.521)	0.604(0.243–1.500)	0.589(0.233–1.492)
Q3	1.656(1.189–2.307)	1.652(1.186–2.302)	1.431(1.010–2.027)	0.828(0.354–1.936)	0.828(0.354–1.941)	0.727(0.304–1.741)
Q4	2.774(1.991–3.864)	2.712(1.944–3.783)	2.054(1.442–2.927)	1.982(0.843–4.662)	1.955(0.830–4.605)	1.470(0.607–3.559)
P for trend	<0.0001	<0.0001	<0.0001	<0.0001	<0.0001	<0.0001

A Model 1: Adjusted for the age, current smoking status, current alcohol drinking status.

B Model 2: Adjusted for the variables in the model 1 plus diabetes, hypertension disease.

C Model 3: Furthered adjusted for the same set of variables in the model 2 plus triglyceride.

### Association between sUA level and NAFLD risk

We examined the associations between the sUA level and the risk of NAFLD by BMI stratification in postmenopausal women (Table 4). The presence of sUA predicted the risk of NAFLD only in nonobese individuals. In model 1, we adjusted for age, current smoking status, and current alcohol drinking status. Compared with individuals in Q1, the ORs (95% CIs) for NAFLD among individuals in Q2-Q4 were 1.527 (1.084–2.152), 1.656 (1.189–2.307) and 2.774 (1.991–3.864), respectively, P value for trend <0.0001. Model 2 was adjusted for the variables in model 1 plus diabetes and hypertensive disease. After adjustment for the above factors, the ORs (95% CIs) for NAFLD among individuals in Q2-Q4 were 1.514 (1.074–2.133), 1.652 (1.186–2.302) and 2.712 (1.944–3.783), respectively, *P* value for trend <0.0001. Model 3 was further adjusted for the same set of variables in model 2 plus TG. After adjustment for the above factors, the ORs (95% CIs) for NAFLD among individuals in Q2-Q4 were 1.518 (1.062–2.169), 1.431 (1.010–2.027) and 2.054 (1.442–2.927), respectively, *P* value for trend <0.0001.

## Discussion

Generally, obesity with a high BMI is a well-established risk factor for NAFLD^6,16–18^. However, as mentioned above, the prevalence of NAFLD in Chinese populations with nonobese individuals has been shown to be high, with a higher prevalence in males than females. Moreover, a large number of studies have indicated that the association between sUA and NAFLD is significantly greater in females than in males. In the present study, the prevalence of NAFLD was 38.8% in females, which might be higher than previously reported^11,12^. Perhaps there was a selection bias, as subjects who participate in general health check-ups are more concerned about their health problems. In this study, we initially hypothesized that there would be some differences in the sUA level between obese and nonobese subjects with NAFLD in postmenopausal females. As a result, several important findings were observed.

First, we found that the sUA level was significantly associated with increased NAFLD risk in females. For subjects with the highest sUA level, the prevalence of NAFLD reached 57.2%. The positive association and dose-response relationship between the sUA level and the presence of NAFLD were higher in the postmenopausal population than in the nonmenopausal population. These results are in agreement with those of previous studies demonstrating that sUA exhibited progressive effects on the development of NAFLD^19–23^. However, most of the studies were conducted in a general population or in obese individuals. We examined the associations between sUA and NAFLD in postmenopausal females and found that regardless of BMI, NAFLD was more prevalent in postmenopausal subjects than nonmenopausal subjects.

Second, in both nonmenopausal and postmenopausal subjects, in the BMI-stratified analysis, the positive association and dose-response relationship between the sUA level and the prevalence of NAFLD were significant in the nonobese population. Previous studies have indicated that not all subjects with NAFLD are obese, especially in East Asian countries^6,16–18^. Furthermore, we found that after controlling for BMI, there was a difference in the course of NAFLD between nonmenopausal and postmenopausal subjects. Postmenopausal subjects seemed to be more likely to have NAFLD than nonmenopausal subjects. This is possibly related to the menopause status and associated hormonal and metabolic changes^24–26^. Postmenopausal women are more susceptible to weight gain, fat redistribution and dyslipidemia, all of which are major hallmarks of metabolic syndrome associated with increased NAFLD risk^27–30^.

Third, we found that higher sUA levels, even within the normal range, were significantly positively and independently associated with increased NAFLD risk, representing a predictive biomarker for NAFLD in nonobese postmenopausal women. Nonobese postmenopausal women with NAFLD are not uncommon. As recently as 2014, a cross-sectional study of 528 normal-BMI postmenopausal women in China showed the same conclusion^25^. In a Jingchang cohort from a Chinese population, the association between the sUA level and NAFLD was stronger in premenopausal women than in postmenopausal women^24^.

The association mechanism between uric acid and NAFLD in postmenopausal women is currently unclear. Population studies have shown that estrogens play a protective role against NAFLD in women^31^. The decrease in estrogens due to the onset of menopause renders postmenopausal women more susceptible to fat redistribution to abdominal areas, weight gain, dyslipidemia, and insulin resistance, which are associated with NAFLD^28^. In addition, some studies have found that premenopausal women’s estrogen levels may promote more effective removal of urate in the kidney^32–34^. What’s more, the mechanisms including hypertension, diabetes, insulin resistance, dyslipidemia, hyperuricemia, and obesity in patients with NAFLD can explain the positive correlation between sUA and NAFLD^35–37^.

However, there were some limitations of our study deserve comment. Firstly, this study was performed in a health check-up population sample of Southwest China women; thus, the findings are likely to be only applicable to health check-up women in Southwest China. Second, although previous reports suggest that female estrogen would affect the serum uric acid levels and indirectly promote the progress of NAFLD^32–34^ as observed, our cross-sectional study design tends to leave uncertainty regarding the temporal sequence of reason-outcome relations. Thus, confirming the relation between menopause and postmenopausal women’s estrogen, sUA levels and incident NAFLD in a prospective longitudinal context would be valuable. Furthermore, it would be deservable to prospectively study if increasing other unmeasured confounding factors.

In conclusions, the positive association and dose-response relationship between the sUA level and the prevalence of NAFLD were significant in the nonobese postmenopausal population. Higher sUA levels can be used as a predictive biomarker for NAFLD in nonobese postmenopausal women.

## Methods

### Subjects

This study used data obtained from subjects who underwent routine health examinations at the Health Management Center of the West China Hospital of Sichuan University from January 2018 through December 2018. The cross-sectional population consisted of 5109 female individuals who underwent abdominal ultrasonography. Those with serologic markers for hepatitis B or C virus, alcohol consumption greater than 140 g/week, known liver disease because of another etiology, history of treatment with exogenous estrogen or tamoxifen, menopausal history due to bilateral ovariectomy, drug use, or radiotherapy, history of medication such as uric acid lowering agent were excluded. Finally, a total of 4323 subjects over 18 years of age were included in this study. Written informed consent was obtained from all participants. The study protocol was approved by the Research Ethics Committee of Sichuan University. All methods in this study were in accordance with the relevant regulations and guidelines.

### Anthropometric, laboratory and ultrasonographic measurements

Anthropometric and laboratory measurements were conducted in the morning after an overnight fast. Medical, smoking and drinking histories were taken by a physician. Waist circumference, height and body weight were measured to the nearest 0.1 cm, 0.1 cm and 0.1 kg, respectively, without shoes or thick clothing. Body mass index (BMI) was calculated as weight in kilograms divided by height in meters squared. Systolic blood pressure (SBP) and diastolic blood pressure (DBP) were measured using an automated sphygmomanometer with the subject in a sitting position in a quiet environment.

The laboratory measurements included sUA, fasting plasma glucose (FPG), total bilirubin (TBIL), direct bilirubin (DBIL), indirect bilirubin (IBIL), high-density lipoprotein cholesterol (HDL-C), low-density lipoprotein cholesterol (LDL-C), triglyceride (TG), total cholesterol (TC), aspartate aminotransferase (AST), alanine aminotransferase (ALT), alkaline phosphatase (ALP), and gamma-glutamyl transpeptidase (GGT). All factors were measured by an immunochemical automated analyzer (Abbott AxSYM) using standard methods.

The ultrasound measurements were performed by the radiologist who was engaged in abdominal ultrasonography.

### Diagnostic criteria

The diagnosis of NAFLD was based on the guidelines for the assessment and management of NAFLD in the Asia-Pacific region, as follows: (1) imaging findings of fatty liver disease; (2) absence of excessive alcohol consumption (ethanol intake <140 g/week for men and 70 g/week for women); and (3) exclusion of diseases leading to steatosis, such as hepatitis C, hepatitis B, alcohol-related liver disease, and haemochromatosis^6^.

In this study, fatty liver disease was recognized by the presence of at least 2 of 3 abnormal findings on abdominal ultrasonography, including diffusely increased liver echogenicity with greater liver echogenicity than kidney or spleen echogenicity, vascular blurring, and deep attenuation of the ultrasound signal. The ultrasonographic outcome was determined by at least two experienced radiologists who were blinded to the laboratory values of the examinees. In addition, fatty liver disease was categorized into three groups according to the results of abdominal ultrasonography (i.e., mild, moderate, or severe).

### Statistical analysis

Statistical analysis was performed with SAS statistical software (version 9.4). Continuous variables are expressed as the mean ± SD, and categorical variables are displayed as percentages (%). For continuous variables, parameters that followed a normal distribution were analyzed by t-test or ANOVA and are expressed as suitable. The chi-square test was used to compare categorical variables between two groups, and the Cochran-Mantel-Henszel test was used to compare categorical variables among multiple groups. Cochran-Armitage trend test was used to compare the prevalence of NAFLD in subjects with different uric acid levels. Multivariate models were used to adjust for confounding variables. Model 1 was adjusted for age, current smoking status, and current alcohol drinking status. Model 2 was adjusted for the variables in model 1 plus diabetes and hypertensive disease. Considering the independence between covariates, Model 3 was further adjusted for the same set of variables in model 2 plus TG. All P values are 2-sided, and a P value of <0.05 was considered statistically significant.

In this study, the sUA data are presented according to sex-specific quartiles. The quartiles in this female population were categorized as follows: Q1 ≤ 230 mmol/L, Q2: 231–270 mmol/L, Q3: 271–310 mmol/L and Q4 ≥ 311 mmol/L. BMI was used as an index of obesity in this study, and the participants were divided into two groups according to BMI (i.e., the nonobese group, at BMI < 25 kg/m^2^, and the obese group, at BMI ≥ 25 kg/m^2^) based on the World Health Organization’s Asia-Pacific guidelines^38^.

## Data Availability

The datasets generated during and/or analyzed during the current study are available from the corresponding author on reasonable request.
